# Prevalence and characteristics of immune checkpoint inhibitor-related myocardial damage: A prospective observational study

**DOI:** 10.1371/journal.pone.0275865

**Published:** 2022-11-15

**Authors:** Tatsuya Nishikawa, Takako Inoue, Tomoyuki Otsuka, Ikumi Kuno, Yoji Kukita, Harumi Nakamura, Yoshihiko Ikeda, Taku Yasui, Wataru Shioyama, Toru Oka, Keiichirou Honma, Kinta Hatakeyama, Hiroshi Miyata, Taiki Isei, Ryu Ishihara, Toru Kumagai, Kazuo Nishimura, Masashi Fujita

**Affiliations:** 1 Department of Onco-Cardiology, Osaka International Cancer Institute, Osaka, Japan; 2 Department of Thoracic Oncology, Osaka International Cancer Institute, Osaka, Japan; 3 Department of Medical Oncology, Osaka International Cancer Institute, Osaka, Japan; 4 Laboratory of Genomic Pathology, Osaka International Cancer Institute, Osaka, Japan; 5 Department of Pathology, National Cerebral and Cardiovascular Center, Suita, Japan; 6 Department of Pathology, Osaka International Cancer Institute, Osaka, Japan; 7 Department of Surgery, Osaka International Cancer Institute, Osaka, Japan; 8 Department of Dermatological Oncology, Osaka International Cancer Institute, Osaka, Japan; 9 Department of Gastrointestinal Oncology, Osaka International Cancer Institute, Osaka, Japan; 10 Department of Urology, Osaka International Cancer Institute, Osaka, Japan; UT MD Anderson Cancer Center, UNITED STATES

## Abstract

An increasing number of patients with cancer are being treated with immune checkpoint inhibitors. Consequently, the incidence of immune checkpoint inhibitor-related myocarditis has been increasing. Nonetheless, the diagnostic criteria for the immune checkpoint inhibitor-related myocarditis have not been sufficiently established. Therefore, the real-world incidence or prevalence of immune checkpoint inhibitor-related myocardial damage remains unknown. This was a single-center cohort study that included 100 patients admitted for immune checkpoint inhibitor therapy for any type of cancer. The patients underwent monthly measurement of cardiac troponin I and N-terminal pro-brain natriuretic peptide levels with electrocardiography. Additionally, echocardiography was performed every 3 months. Our protocol was continued until 6 months after the initiation of immune checkpoint inhibitors. We defined immune checkpoint inhibitor-related myocardial damage as an increase in cardiac troponin I levels by >0.026 ng/mL and/or a decrease in the left ventricular ejection fraction by >10% to <53% on echocardiography. The mean patient age was 64 years; 71% were men. The most commonly used immune checkpoint inhibitor was nivolumab (47%), followed by pembrolizumab (29%). Overall, 5% of patients received combination therapy. Among 100 patients, 10 (10%) were diagnosed with immune checkpoint inhibitor-related myocardial damage. Among them, five patients underwent endomyocardial biopsy. Of these patients, four were histopathologically observed to have lymphocyte infiltration in their myocardium. In conclusion, serial cardiac troponin I measurement during immune checkpoint inhibitor treatment could help detect early-phase myocardial damage. The prevalence of myocardial damage was much higher than previously expected.

## Introduction

The prognosis of various cancers has improved owing to the development of novel anticancer drugs categorized as immune checkpoint inhibitors (ICIs) [1]. Recently, the number of patients with cancer eligible for ICI treatment has considerably increased [2]. In addition to ICI use, the incidence of immune-related adverse events (irAEs) has become a growing issue in clinical settings. Among the irAEs, myocarditis is very rare, with an incidence rate of 0.09%–1.14% [3, 4]; however, it is potentially lethal. Although clinically relevant myocarditis is rare, it cannot be ignored owing to the increased number of patients with cancer receiving ICI treatment. The standard definition for the diagnosis, pathophysiological mechanism, management, and prevention of ICI-related myocarditis reported in previous studies, such as case reports, reviews, and registry studies, is yet to be clearly established. Therefore, prospective data are required to provide evidence. Herein, we prospectively assessed the prevalence, characteristics, and prognosis of myocardial damage in patients treated with ICIs.

## Materials and methods

### Patients

The study protocol and consent forms were approved by the local ethics committee of Osaka International Cancer Institute (approval no. 19189). All patients provided written informed consent prior to their inclusion in the prospective study.

In this prospective cohort study, we comprehensively screened consecutive patients with any type of cancer scheduled for admittance to our hospital with the help of a medical clerk between March 2020 and September 2020 to initiate ICI treatment based on the clinical state. As the present study involved all the clinical departments in our institute, it was challenging to collect enough samples prospectively. Therefore, as a pilot study, we set the sample size at approximately 100 patients. Overall, 107 patients who provided informed consent were enrolled in this study. Among them, we excluded five patients who dropped out of the study by their requests and two patients who had missing data for cardiac troponin I (cTnI) during the follow-up period. Among the remaining patients, we included three patients who missed undergoing either electrocardiography or echocardiography once during follow-up because they did not have any signs of myocarditis. Finally, 100 patients were enrolled in this study. Case numbers are detailed in all figures and tables.

### Study protocol

The enrolled patients underwent measurements for the cTnI and N-terminal pro-brain natriuretic peptide (NT-proBNP) levels and electrocardiography before ICI therapy initiation, and they were observed on follow-up once a month. Echocardiography was performed once before ICI therapy initiation and subsequently every 3 months. Patients were observed on follow-up for 6 months after ICI therapy initiation. The permissive error was 1 week for the first month, 2 weeks for the second and third months, 3 weeks for the fourth month, 4 weeks for the fifth month, and 6 weeks for the sixth month, depending on the clinical course of each patient receiving ICI. Patients were observed during follow-up until the sixth month, even if ICI therapy was discontinued for any reason. However, follow-up was discontinued when patients died or stopped treatment to transition to palliative care or the best supportive care. Therefore, cancer prognosis, especially for less than 6 months, could be potentially biased. Echocardiography data were obtained using an IE 33 imaging device (Philips Healthcare, Amsterdam, The Netherlands). Left ventricular ejection fraction (LVEF) was measured using the modified Simpson method. Global longitudinal strain (GLS) was assessed using QLAB15 (Philips). For patients who were clinically suspected of having ICI-related myocarditis, cardiac magnetic resonance (CMR) imaging (Aera 1.5 T, Siemens Healthineers, Munich, Germany) and/or endomyocardial biopsy (EMB) was performed if medical conditions were suitable.

### Definition of ICI-related myocardial damage and myocarditis

We defined “ICI-related myocardial damage” as an increase in the cTnI levels and/or a decrease in the LVEF. The cut-off value for cTnI was set at >0.026 ng/mL in accordance with the manufacturer’s reference (cutoff value, 26.2 pg/mL; ARCHITECT STAT high-sensitivity troponin I, Abbott Laboratories, Chicago, IL, USA). The other definition was a decrease in the LVEF by >10% to <53% on echocardiography [5, 6]. NT-proBNP and electrocardiography were used for supportive assessments. The definition of ICI-related myocarditis has not yet been fully established; thus, the definition was not consistent with that reported in previous studies [4, 7–10]. For comparison, we used the position statement for myocarditis from the European Society of Cardiology (ESC) [8], the new definition of ICI-related myocarditis proposed by Bonaca et al. [7], ICI-related myocarditis by the American Society of Clinical Oncology (ASCO) guidelines [11], and common terminology criteria for adverse event (CTCAE) grading [12]. Both definite and clinically suspected myocarditis cases were counted as myocarditis for the ESC position statement. According to Bonaca’s definition, pathological evidence is based on abnormal lymphocyte infiltration in the myocardium.

### Immunohistochemical staining of cardiomyocytes

Buffered formalin-fixed and paraffin-embedded (FFPE) tissues obtained from endomyocardial biopsies were subjected to immunohistochemistry staining. FFPE tissues were cut into 4-μm-thick sections and stained with hematoxylin, eosin, and Masson’s trichrome stains for morphological observation. The immunohistochemical study was performed using primary antibodies against CD3 (#N1580, Dako, Glostrup, Denmark) for T lymphocytes, CD4 (NCL-L-CD4-368, Leica Biosystems, Wetzlar, Germany) for helper T lymphocytes, CD8 (NCL-CD8-4B11, Leica Biosystems) for cytotoxic T lymphocytes, and CD68 (M0876, Dako) for macrophages. In addition, a commercially available tenascin-C antibody (4F10TT, #10337, Immuno-Biological Laboratory Co., Ltd., Gunma, Japan) was also used. We used BOND-III automated immunohistochemical staining system (Leica Microsystems K.K., Tokyo, Japan). After immunostaining, CD3-, CD8-, and CD68-positive cells were counted by two pathologists in a high-power field in the most intensively lymphocyte-infiltrated area. Each sample was classified using the grading system reported by Palaskas et al. [13].

## Results

### Patients’ baseline characteristics

**Fig 1:**
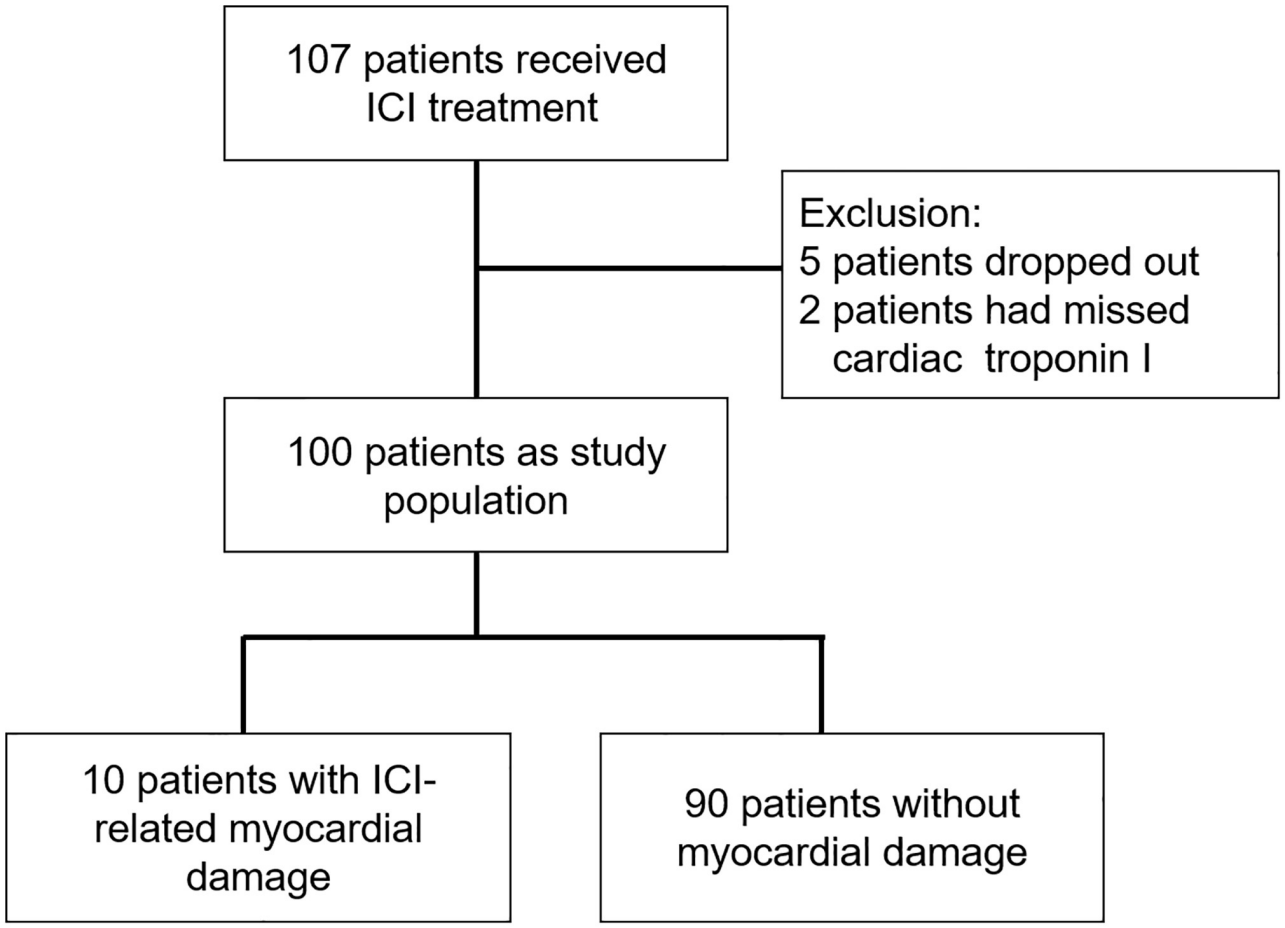
Flowchart of the patient selection process.

Overall, 100 patients were included, and their data were analyzed (Fig 1). Patients’ characteristics are shown in Table 1. The mean patient age was 64 years, and 71% were men. The most commonly used ICI was nivolumab (47%), followed by pembrolizumab (29%). Overall, 5% of patients received combination therapy. The most frequent types of cancer were lung cancer, head/neck cancer, esophageal cancer, and malignant melanoma. Among 28 patients with lung cancer, three had small cell cancer, whereas the others had non-small cell cancer. None of the study participants had a history of congestive heart failure. However, one patient had a history of myocardial infarction without reduced LVEF. Tyrosine kinase inhibitors (TKIs) or vascular endothelial growth factor inhibitors (VEGFIs) were used for cancer treatment before ICI treatment for five patients (S1 Table in S1 File). In addition, the patients who underwent ICI therapy with concomitant chemotherapy are listed in S2 Table in S1 File. Among the 21 patients, five used TKIs or VEGFIs. All patients had normal cTnI levels before ICI therapy initiation. After ICI treatment, 29% had irAEs in any organ and grade. The average follow-up period was 144 (median, 168; interquartile range (IQR), 112–175) days owing to death or transition to palliative care.

**Table 1:** Baseline characteristics of patients.

All patients (n = 100)			
Age (years)	64±14	Past history	
Sex		Congestive heart failure	0
Male	71	Stroke	6
Female	29	Atrial fibrillation	5
Body mass index (kg/m^2^)	21±3	Hypertension	36
History of smoking	77	Diabetes mellitus	15
ECOG performance status		Dyslipidemia	23
0	43	Chronic kidney disease	11
1	47	Ischemic heart disease	3
2	9	Myocardial infarction	1
3	1	Percutaneous coronary intervention	1
ICI		Autoimmune disease	0
Nivolumab	47	Thoracic radiotherapy	31
Pembrolizumab	29	Response to ICI	
Durvalumab	10	Progressive disease	45
Atezolizumab	8	Stable disease	29
Avelumab	1	Partial response	9
Ipilimumab + nivolumab	5	Complete remission	1
Combined use with ICI	21	N/A	16
TKI/VEGFI	7	History of cardio-toxic therapy	
Other anti-cancer drugs	14	Anthracycline	4
Cancer type		TKI/VEGFI	5
Lung	28	HER2 inhibitor	2
Small cell cancer	3	Prevalence of irAEs	29
Non-small cell cancer	25	Hepatitis	5
Head/neck cancer	22	Thyroiditis	3
Esophageal cancer	21	Adrenal insufficiency	3
Malignant melanoma	12	Arthritis	3
Kidney	9	Cerebral meningitis	3
Gastric	3	Nephritis	1
Breast	2	Cytokine release syndrome	1
Bladder	1	Others	10
Uterus	1	Steroid therapy for irAE	13
Unknown	1	Follow-up period (day)	144±52
Metastatic/recurrent cancer	96	Death during the follow-up period	25
Adjuvant therapy	4		
Medication at initiation of ICI			
Statin	13		
ACEi/ARB	17		
β-blockers	5		
Diuretics	2		

ACEi, angiotensin-converting enzyme inhibitor; ARB, angiotensin II receptor blocker; ECOG, Eastern Cooperative Oncology Group; HER2, human epidermal growth factor receptor; ICI, immune checkpoint inhibitor; irAE, immune-related adverse event; TKI, tyrosine kinase inhibitor; VEGFI, vascular endothelial growth factor inhibitor.

### Prevalence of ICI-related myocardial damage and myocarditis

Based on our definition, we determined that ICI-related myocardial damage occurred in 10% (10/100) of our population, and the prevalence was 0.014 cases per person-month. We performed EMB in five patients with ICI-related myocardial damage. As a result, 4% of cases (4/100) were histopathologically diagnosed with myocardial inflammation. We identified 10 patients showing an increase in the cTnI levels and/or a decrease in the LVEF (Tables 2 and 3). We diagnosed them with “ICI-related myocardial damage.” EMB and CMR imaging were performed if clinically and physically possible. Some patients could not undergo these examinations owing to unconsciousness, patient refusal, or limited medical resources. As shown in Table 3, CMR were performed in four of 10 patients with ICI-related myocardial damage.

**Table 2:** Characteristics of cancer, ICI treatment, and myocardial damage of the patients with ICI-related myocardial damage.

Case	Sex	Age	ICI	Cancer type	Best response	concomitant drug	Comorbidities	ICI initiation to myocardial damage [days]	discontinuation of ICI before myocardial damage
1	M	54	Ipi+ Nivo	Kidney	SD	-	HTN, DM, DLp	65	-
2	M	47	Pembro	Kidney	PR	Axitinib	-	142	+ (irAE)
3	M	82	Durva	Lung (non-small cell)	SD	-	HTN, DM	159	+ (PD)
4	M	66	Pembro	Kidney	SD	Axitinib	-	44	-
5	M	76	Pembro	Cancer of unknown primary (MSI-high)	SD	-	-	43	-
6	F	83	Nivo	Malignant melanoma	PD	-	HTN, DLp	220	-
7	M	66	Pembro	Esophageal	NA	-	HTN	12	-
8	M	74	Nivo	Larynx	CR	-	HTN	209	-
9	F	71	Pembro	Oral cavity	PD	-	-	81	-
10	M	57	Pembro	Oropharinx	PR	CDDP 5FU	-	83	-

**Fig 2:**
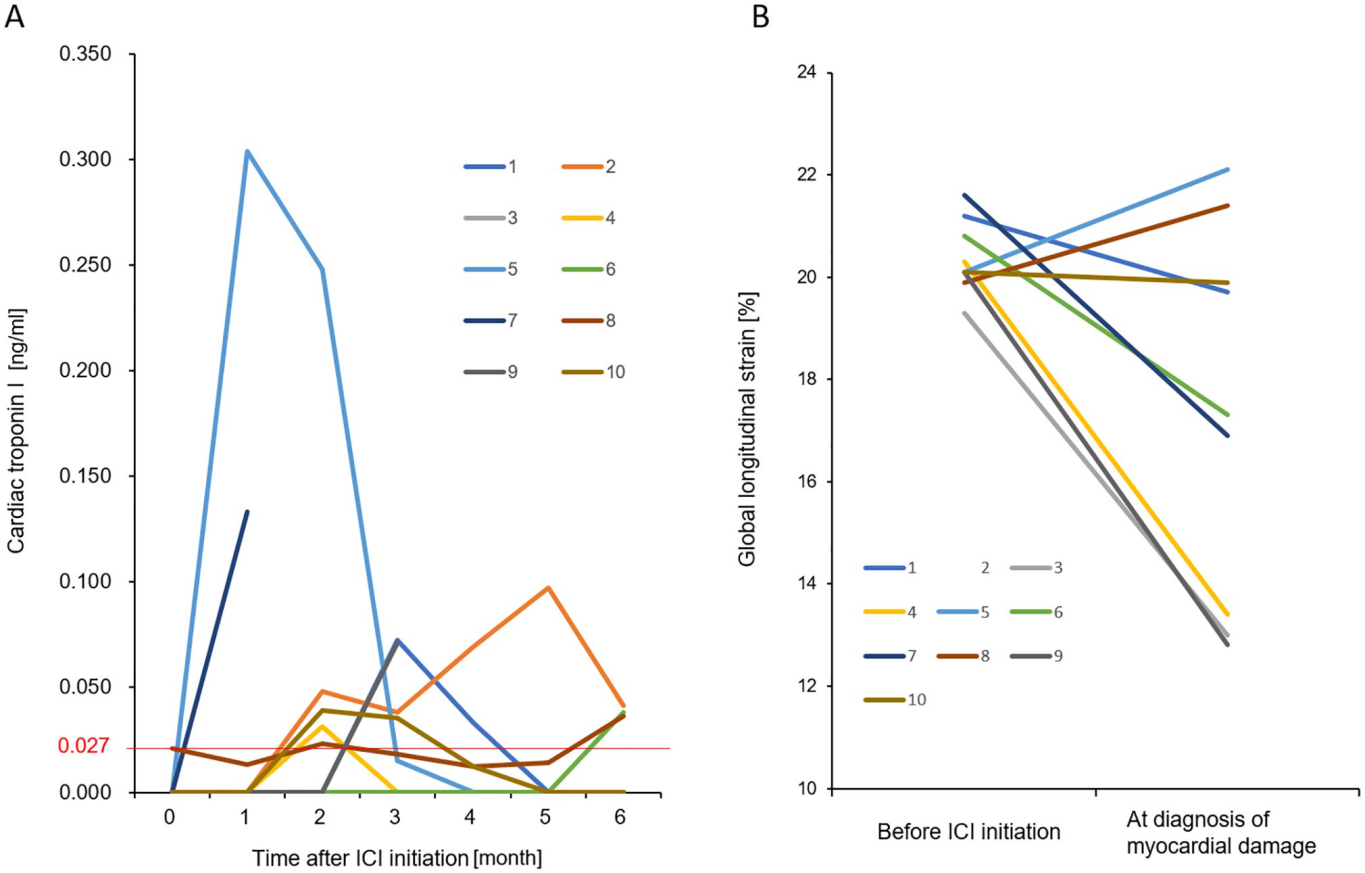
**(A) cTnI Levels During Follow-up in Patients With ICI-related Myocardial Damage.** Among the 10 patients with ICI-related myocardial damage, four showed sufficient serial follow-ups after an increase in cardiac troponin I levels. In Cases 1, 5, and 10, cardiac troponin I level decreased only after discontinuation of ICI therapy. In Case 2, cardiac troponin I level increased even under steroid and immunosuppressive drugs for other critical irAEs. In addition, the cardiac troponin level remained high for several months. The case numbers correspond with those in Tables 2–4 and Fig 2. Abbreviations: cTnI, cardiac troponin I; ICI immune checkpoint inhibitors; irAEs, immune-related adverse events. **(B) Change in GLS From Baseline to the Point of Myocardial Damage.** Among 10 patients with ICI-related damage, five showed a reduction in GLS by >15%, as compared with the baseline. Case 2 is not presented because echocardiography did not have sufficient quality for GLS calculation.

CR, complete remission; DLp, dyslipidemia; DM, diabetes mellitus; F, female; HTN, hypertension; ICI, immune checkpoint inhibitor; M, male; PD, progressive disease; PR, partial remission; SD, stable disease. The case numbers correspond with those in Tables 3 and 4, Figs 1 and 2.

**Table 3:** Clinical presentation, diagnosis, and grading of the patients with ICI-related myocardial damage.

Case	Symptom	Echocardiography	Rechallenge with ICI*	Treatment for myocardial damage	Survival / outcomes of myocardial damage	Co-occurrence of other irAEs	CMR	EMB	Bonaca’s	ESC	ASCO
1	none	NC	-	-	alive/ cured	myositis	T2 neg. LGE n/a	pos.	Definite	Definite	G1
2	none	PE	-	Steroid + IST	alive/ cured	hepatitis, CRS	T2 neg. LGE neg.	pos.	Definite**	Definite**	G1
3	pleural effusion	reduced EF, PE	-	-	alive/ cured	-	-	pos.	Definite**	Definite**	G2
4	dyspnea	reduced EF, PE	+		alive/ cured	-	T2 neg. LGE neg.	pos.	Definite**	Definite**	G3
5	none	NC	-	-	alive/ cured	T1DM, thyroiditis	T2 neg. LGE neg.	neg.	Subclinical	clinically suspected	G1
6	fatigue	reduced EF, PE	-	-	died due to pneumonia/ NA	adrenal insufficiency, suspected myositis	-	-	Probable	clinically suspected	G1
7	fatigue	reduced EF, PE	-	-	died due to other irAEs / NA	meningitis	-	-	Probable	clinically suspected	G3
8	none	NC	+	-	alive/ cured	adrenal insufficiency	-	-	Subclinical	unsatisfied criteria	G1
9	none	NC	-	-	died due to cancer/ NA	-	-	-	Subclinical	unsatisfied criteria	G1
10	none	NC	no ICI cessation	-	alive/ cured	hepatitis, thyroiditis	-	-	Subclinical	unsatisfied criteria	G1

*Retreatment with ICI after stopping ICI due to myocardial damage.

** Tissue pathology diagnostic myocarditis (S5 Table in S1 File) included satisfaction of only the immunohistochemical criteria of lymphocyte infiltration, but without degeneration and/or necrosis of cardiomyocytes. These were determined as borderline myocarditis in the Dallas criteria [14] and chronic myocarditis in the World Heart Federation criteria [15].

ASCO, American Society of Clinical Oncology; ICI, immune checkpoint inhibitor; CMR, cardiac magnetic resonance; EF, ejection fraction; EMB, endomyocardial biopsy; ESC, European Society of Cardiology; G0–3, Grades 0–3; GLS, global longitudinal strain;; irAE, immune-related adverse event; IST, immunosuppressive therapy; LGE, late gadolinium enhancement; n/a, not available; NC, no change; Neg., negative; PE, pericardial effusion; Pos., positive; T1DM, type 1 diabetes mellitus. The case numbers correspond with those in Tables 2 and 4, Figs 1 and 2.

**Table 4:** Characteristics of histopathological investigation of endomyocardial biopsy samples in patients with ICI-related myocardial damage.

Case	Myocyte necrosis/degeneration	local fibrosis	CD3 count (/HPF)	CD8 count (/HPF)	CD68 count (/HPF)	PD-L1	TNC	Palaskas’ grading system
1	+	+	40	20	5	+	+	Group 2
2	-	+	8	13	5	-	+	Group 1A
3	-	+	41	18	16	-	+	Group 1B
4	-	+	18	6	24	-	+	Group 1B
5	-	+	2	2	9	-	+	Group 0

HPF, high-power field; PD-L1, programmed death-ligand 1; TNC, tenascin-C.

The case numbers correspond with those in Tables 2 and 3, Figs 1 and 2.

In the present study, a total of four patients had a history of Adriamycin treatment (Table 1), and they were patients without ICI-related myocardial damage. Adriamycin, unlike the other cardiotoxic chemotherapeutic drugs, is known to cause irreversible cardiac damage even in late-onset with increase in the cardiac troponin I/T levels. Therefore, if we had excluded the four patients, the prevalence of ICI-related myocardial damage would have been 10.4% (10 of 96 patients).

According to the results, the prevalence of ICI-related myocarditis was assessed to be 7% as per the ESC criteria (S3 Table in S1 File), 3% as per CTCAE Grade ≥2 (S4 Table in S1 File). Based on the ASCO guidelines (S5 Table in S1 File), cardiac toxicity was considered to have occurred in 10% of patients. Furthermore, according to Bonaca’s criteria (S6 Table in S1 File), 4% of cases were definite, 2% were probable, and 4% were subclinical myocarditis. The prevalence of total ICI-related myocardial damage for each ICI was 3% (1/47) for nivolumab, 14% (4/29) for pembrolizumab, 11% (1/9) for durvalumab, and 20% (1/5) for combination therapy when diagnosed using Bonaca’s criteria. However, none of the patients died due to cardiovascular events related to myocarditis.

As shown in Table 3, among the 10 patients with myocardial damage, seven cases co-occurred with other irAEs during follow-up. Only two patients exhibited atypical ST-T changes in electrocardiography findings, but these were not the characteristic broad ST-T changes that are usually observed in typical myocarditis. Other arrhythmias, such as atrioventricular block, atrial fibrillation, or ventricular arrhythmia, were not observed. In half of the patients, response to ICI indicated stable disease or complete remission. None of them had received anthracycline before and after ICI treatment. Furthermore, the duration between ICI initiation and myocarditis ranged from 12 to 209 days (median, 82 days).

### Characteristics of patients with ICI-related myocardial damage

ICI myocarditis was primarily observed in men and was caused by nivolumab, pembrolizumab, durvalumab, and combination therapy. The type of cancer also varied, and cancer responses to ICI therapy were not consistent. Approximately, only half of the patients were evaluated as having stable disease. Five patients had hypertension, two had diabetes mellitus, and two had dyslipidemia. None of them had a history of potentially cardiotoxic treatment, such as anthracycline, TKI, HER2 receptor inhibitor, or thoracic radiation therapy. Additionally, none of the patients had a history of heart failure.

Serial changes in the cTnI levels during follow-up are shown in S7 Table in S1 File and Fig 2A. The follow-up of Cases 7 and 9 was discontinued because of the discontinuation of cancer therapy and transition to palliative care, respectively. In seven cases, the cTnI levels increased within 3 months but decreased within 2 months after discontinuing ICI therapy. In Case 2, cardiac damage was observed after administering steroid and immunosuppressant drugs for severe non-cardiac irAEs (Tables 2 and 3). The levels of cTnI had remained high but had slightly decreased when prednisolone and two immunosuppressant drugs were administered orally for several months. In fact, only one patient showed no increase in the cTnI levels (Case 3) but exhibited a decrease in LVEF from 57% to 41% and an increase in the NT-proBNP levels (S8 and S9 Tables in S1 File). In contrast, Case 4 showed typical changes in clinical measurements with an increase in the cTnI and NT-proBNP levels and a decrease in the LVEF. Therefore, as shown in S1 and S2 Figs in S1 File, the LVEF and NT-proBNP levels did not consistently change with cTnI changes. GLS decreased by >15% from baseline in five patients (Cases 3, 4, 6, 7, and 9); nonetheless, two of these patients (Cases 3 and 4) were accompanied with reduced LVEF (Fig 2B and Table 3). Furthermore, we investigated CMR imaging in only four patients (Cases 1, 2, 4, and 5). All of them had increased cTnI levels, and three had low-grade myocarditis, as defined by EMB; however, none showed significant signs of inflammation, such as positive T2-weighted image and late gadolinium enhancement.

Among the 10 patients with ICI-related myocardial damage, we further assessed the cancer cells of those with sufficient cancer pathological samples obtained by biopsy or surgical resection. The total RNA of cancer tissues was extracted from FFPE samples. As control individuals, we included 14 patients who had enough surgical samples for the assay (S10 Table in S1 File). We performed RNA sequencing by next-generation sequencing to assess the relative expression of all known genes. Among all the genes, we analyzed those that were reportedly related to myocardial diseases. Cancer samples showed myocardial or muscle-related gene expression. As representative genes (*TNNI3*, *MYH6*, *MYH11*, *MYBPC3*, *TNN*, *DMD*, and *DES*), we observed various gene expression patterns in each patient in both the myocardial-damaged group and the control group (S3 Fig in S1 File). There were no highly expressed muscle-related genes in all patients with myocardial damage (M1–5), as compared with the control group (C1–14). Furthermore, we performed heat map analysis for the control and myocarditis groups. Most of them were genes encoding long noncoding RNAs or pseudogenes (S4 Fig in S1 File).

### Characteristics of histopathological study

**Fig 3:**
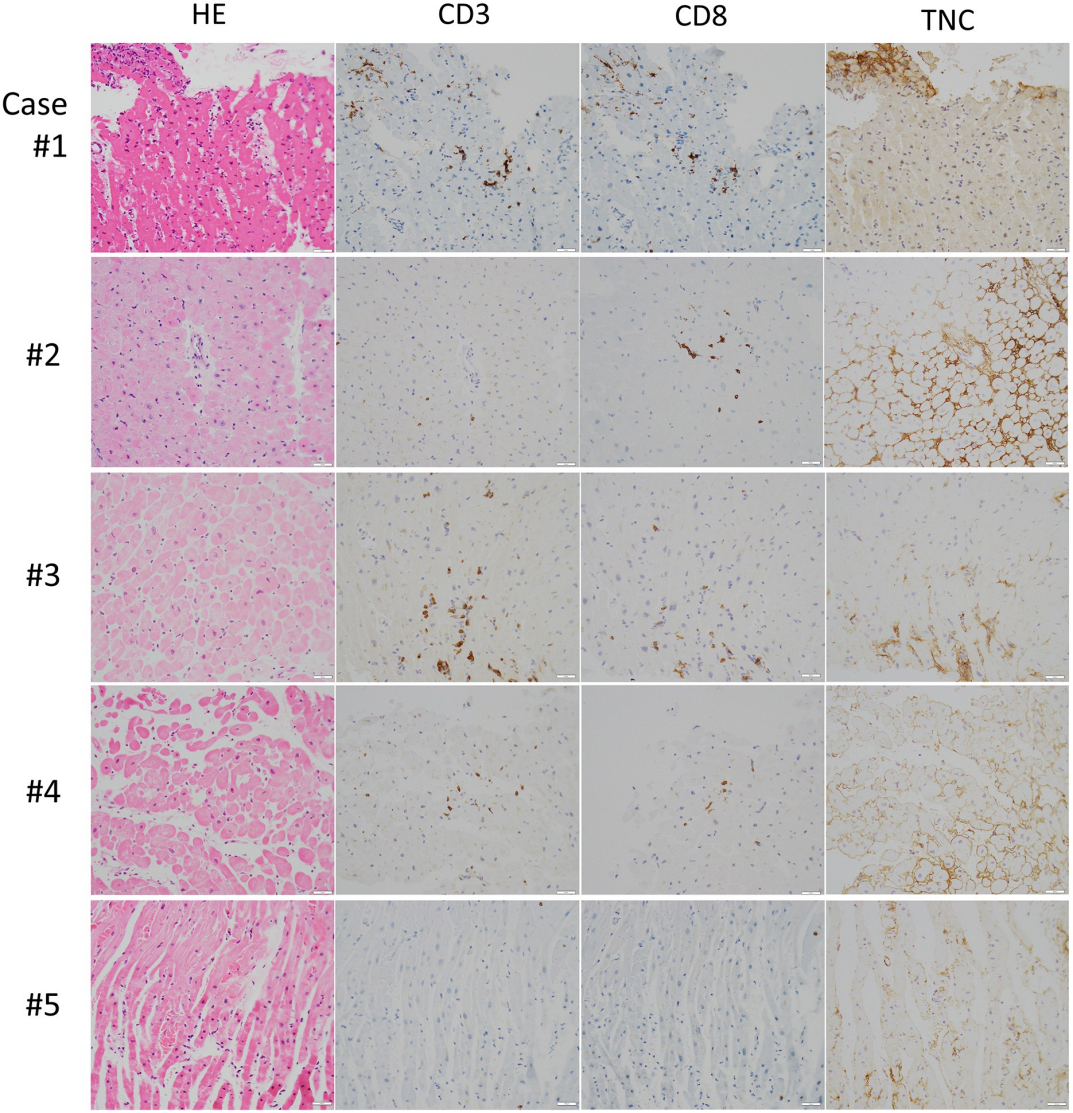
Histopathological study in patients with ICI-related myocardial damage. The image shows immunohistochemical staining of CD3 for T lymphocytes, CD8 for cytotoxic T lymphocytes, and tenascin-C for inflammation in the myocardium. Cases 1–4 showed focal mild T lymphocyte infiltration. All cases showed positive staining for tenascin-C. Abbreviation: ICI immune checkpoint inhibitors. The case numbers correspond with those in Tables 2–4 and Fig 1.

EMB was performed in five out of the 10 patients with ICI-related myocardial damage. We assessed the myocardial infiltration of CD3- and CD8-positive cells and CD68-positive macrophages. In addition, PD-L1 and tenascin-C were immunohistologically assessed (Fig 3 and S5 Fig in S1 File). The results indicated that four out of five patients had mild but focal lymphocyte infiltration, which we diagnosed as low-grade myocarditis (Table 4). PD-L1 was positive only in the most active myocarditis case (Case 1; S5 Fig in S1 File). However, local fibrosis and tenascin-C were interestingly positive in all five patients. Only one patient (Case 1) had myocyte necrosis and degeneration. The newly developed grading system is presented in Table 4.

Definition of Palaskas’ grading systems [13] are as follows: Grade 0, negative; Grade 1, multifocal inflammatory infiltrates without overt cardiomyocyte loss by light microscopy; Grade 1A, mild inflammatory cell score by immunohistochemistry (10–20 inflammatory cells/high-power field); Grade 1B, at least moderate inflammatory cell score by immunohistochemistry (>20 inflammatory cells/high-power field); and Grade 2, multifocal inflammatory cell infiltrates (>40 inflammatory cells/high-power field) with overt cardiomyocyte loss by light microscopy.

## Discussion

To the best of our knowledge, this is the first study to prospectively assess the prevalence of ICI-related myocardial damage, defined as an increase in the cTnI levels and/or a decrease in the LVEF. Furthermore, this single-center study has strength in that our study prospectively enrolled all types of cancer. We found that the prevalence of low-grade myocarditis was much higher than what was expected, although the definition of myocarditis has not been fully established. Serial cTnI measurement could help in detecting low-grade or very early ICI-related myocardial damage; most cases of ICI-related myocardial damage were improved only by discontinuation of ICI therapy, and most patients with ICI-related myocardial damage had other irAEs. We believe that our findings could serve as a basis for future research on ICI-related myocarditis in terms of real prevalence, diagnosis, immunological factors, and management of myocarditis.

### Diagnosis and definition of ICI-related myocarditis

The greatest problem is that the definition for the diagnosis of ICI-related myocarditis has not been fully established [16]. Previous studies [4, 10] have defined myocarditis using a position statement for myocarditis from the ESC [8], which was published in 2013. Another major definition for ICI-related cardiovascular toxicity, including myocarditis, was determined by the ASCO clinical practice guidelines in 2021 [11]. A further study [9] used the criteria proposed by Bonaca et al. as a new definition for ICI-related myocarditis in 2019 to be applied in clinical trials [7]. The definition by the ESC was not always suitable for ICI-related myocarditis because it defines only “histopathologically proven myocarditis” and “clinically suspected myocarditis.” The latter was diagnosed according to symptoms, biomarkers, and CMR imaging. However, according to Zhang et al., caution should be exercised regarding the reliability of the diagnosis for the exclusion of ICI-related myocarditis by CMR imaging [10]. In contrast, the definition proposed by Bonaca et al. is very specific and well established; however, it is designed for future clinical research but not for clinical use. Therefore, it is necessary to establish additional evidence by using a specific definition, which should later be reflected in the oncologists’ guidelines as simpler diagnostic criteria. EMB is supposedly the most invasive but highly specific examination for ICI-related myocarditis. The criteria for myocarditis had been mostly dependent on the classical Dallas criteria, which require myocardial necrosis or degeneration in the histopathological study to satisfy the criteria for active myocarditis. However, recently, low-grade focal lymphocyte infiltration has been frequently observed [13, 17]. Therefore, the definition or grading of histopathology in this field has been evolving [13].

As shown in the present study, an increase in the cTnI levels from baseline should be suspected or considered a sign of myocardial damage regardless of the presence of symptoms or obvious abnormality detected by electrocardiography, echocardiography, and CMR imaging [4, 18]. As indicated in Cases 6 and 7, in the real-world clinical situation, some patients were not suitable for CMR imaging and EMB owing to the impairment of consciousness by ICI-related encephalomeningitis, severe metastatic brain tumor, severe infection, and end of cancer therapy, as also pointed out in a recent report [19]. Furthermore, CMR imaging could not detect low-grade myocarditis in the present study. In addition, NT-proBNP and LVEF provided supporting information on myocardial damage; however, they appeared better when used in combination with cTnI. In the present study, GLS may be a better tool for assessing early myocardial damage than NT-proBNP and LVEF. Therefore, as reported in previous studies, the non-invasive measurement of the cTnI levels is the most sensitive marker for ICI-related myocarditis [4, 18].

### Prevalence of ICI-related myocardial damage

In our study, among 100 patients with cancer who received ICI treatment, 10 showed an increase in the cTnI levels and/or a reduction in the LVEF. None of the patients required hospital admission for heart failure. Had they not been included in our study, they might not have been diagnosed with myocarditis. However, some patients might have developed fulminant myocarditis if cTnI measurement had not been performed precisely on the follow-up to detect early signs of myocarditis. Another important finding of the present study was that four out of five potential patients with myocarditis with EMB had low-grade myocarditis. Recently, a new grading system for ICI-related myocarditis was reported [13]. In this classification system, the histopathological samples were grouped as “definite myocarditis (Group 2),” “myocardial inflammation (group Ia and Ib),” and no myocarditis (Group 0). Among patients with ICI-related myocarditis, low-grade myocarditis is common according to histopathological assessment [13, 17]. In that report, low-grade myocarditis reportedly had a relatively better prognosis than high-grade myocarditis. In our study, all the cases were of low grade, and myocarditis was mostly cured by steroid therapy or by discontinuation of ICI therapy. Interestingly, tenascin-C, which is a sensitive marker for myocardial inflammation [20], stained positively through immunostaining in all five cases. This low-grade myocarditis might sometimes be challenging to diagnose because potential sampling errors may occur. Hence, immunostaining of tenascin-C could provide supportive evidence for myocarditis.

In this study, the patients with ICI-related myocardial damage were mostly male individuals. The possible reason is that the cutoff value for the normal range of cardiac troponin I levels is higher for male than for female individuals. However, all male patients with ICI-related myocardial damage had troponin I levels below our cutoff value at the initiation of ICI treatment, while these levels increased during the study period. Therefore, the sex difference was no conclusive in our study.

### Characteristics of patients with ICI-related myocardial damage

In the present study, in patients with ICI-related myocardial damage, this often co-occurred with other irAEs. Kichenadasse et al. reported that 19.8% of patients with irAEs due to atezolizumab had multiorgan irAEs [21]. In addition, Shimozaki et al. reported that 108 of 212 patients who received anti-PD-1/PD-L1 antibody therapy had experienced irAEs and 42 (39%) had multiorgan irAEs [22]. Further, Shankar et al. reported that 9.6% of patients treated with anti-PD-1/PD-L1 antibody had multiorgan irAEs, whereas 28% of those had some irAEs [23]. According to this evidence, multiorgan irAEs are not rare, although it is unknown whether there is a common mechanism underlying multiorgan irAEs. However, according to a study conducted by Johnson et al., ICI-related myocarditis and myositis can occur by the same mechanisms [3], and this has been stated in many case reports [24]. In real-world clinical practice, information regarding other irAEs may support a myocarditis diagnosis. A systematic review of reported cases of ICI-related myocarditis showed that the most common irAEs co-occurring with myocarditis were myositis (29%), hepatitis (21%), and thyroiditis (12%), although they occurred in a biased group of patients [24]. In the present study, Cases 6 and 7 were not suitable for either EMB or CMR imaging because these cases exhibited disruption of consciousness; however, they showed a slight increase in the cTnI levels and unbalanced high creatinine kinase levels, suggesting the presence of myositis.

In fact, five possible pathophysiological mechanisms of irAEs have been reported [25]. Among them, myocarditis is considered to be caused by cross-antigen reactivity [3]. To our knowledge, only one previous report demonstrated the potential presence of shared antigens in cancer tissues [3]. In the two reported cases, the patients had ICI-related myocarditis with myosis, which is considered to be the most typical case of shared antigen-associated irAEs. The study reported that the potential muscle-specific genes expressed in cancer cells were *TNNI1*, *TNNT1*, *DES*, *TRDN*, *MYH6*, *MYH11*, and *MYH13*. Herein, we investigated the gene expression in cancer tissues obtained from the study patients. The results showed that some cardiomyocyte-related genes were expressed in cancer cells in patients with myocardial damage (M1–5), and the expression was relatively higher than that in other control individuals; however, each patient with myocardial damage had a completely different gene expression pattern. Furthermore, in the representative genes shown in S3 Fig in S1 File, formerly reported genes [3] did not show high expression in our cancer tissue samples. In summary, as ICI-related myocarditis involves the expression of various clinical phenotypes, the difference in shared antigens in each case might be among the relevant factors for the clinical course. Further prospective research is needed in this area.

### Management of ICI-related myocardial damage

There has been an increasing evidence supporting immunosuppression therapy for fatal fulminant myocarditis or definite myocarditis [9, 13, 26]. However, the management of probable or possible myocarditis remains unknown. The current ASCO guidelines [11] recommend holding ICI even for Grade 1 myocarditis with elevation of only biomarker levels without any symptoms. As shown in the present study, discontinuation of ICIs improved most of the ICI-related myocardial damage. In contrast, there is a possibility of rechallenging ICI after low-grade myocarditis or other irAEs [27, 28]. Further clinical investigations are needed to rechallenge ICIs, particularly for myocarditis.

### Limitations

Our study had some limitations. First, as the present study had a small number of samples, we could not statistically analyze the risk factors or prognosis. For the diagnosis of ICI-related myocardial damage, we included approximately the half of patients who could have EMB and CMR because of the clinical or social situation. In addition, the follow-up period had been set as 6 months; late-onset myocarditis could have been observed after the follow-up period. Furthermore, in the present study, the effects of concomitant chemotherapy and/or TKI/VEGFI in addition to ICI were not thoroughly assessed.

## Conclusion

Serial cTnI measurement during cancer treatment with ICIs could aid in the detection of low-grade myocarditis or early-phase myocardial damage. The prevalence of myocardial damage was much higher than expected in previous studies. For most patients, the increase in the cTnI levels was attenuated by the discontinuation of ICI therapy. Although it is essential to recognize that the increase in cTnI levels could reflect the beginning of ICI-related myocardial damage, it is more important to treat patients through close cooperation between oncologists and cardio-oncologists.

## Supporting information

S1 File (DOCX)
