# Supplementary material for: Prevalence and characteristics of immune checkpoint inhibitor-related myocardial damage: A prospective observational study
Source: PLoS One. 2022 Nov 15;17(11):e0275865. doi: 10.1371/journal.pone.0275865 (PMC9665386; doi:10.1371/journal.pone.0275865)
Supplement: S1 File — (DOCX) [file pone.0275865.s001.docx]

**Supporting information**

**S1 Table. List of TKIs/VEGFIs Used in Patients Without Myocardial Damage Before ICI Initiation.**

|  | **Cancer type** | **TKIs/VGEFIs** |
| --- | --- | --- |
| 1) | Lung (non-small cell) | Alectinib, lorlatinib, ceritinib |
| 2) | Malignant melanoma | Dabrafenib + trametinib |
| 3) | Lung (non-small cell) | Gefitinib, osimertinib, bevacizumab |
| 4) | Kidney | Axitinib |
| 5) | Kidney | Axitinib, sunitinib |

ICI, immune checkpoint inhibitor; TKI, tyrosine kinase inhibitor; VEGFI, vascular endothelial growth factor inhibitors.

**S2 Table. Patients who Received ICI Therapy With Concomitant Chemotherapy.**

|  | **Cancer type** | **Concomitant chemotherapy** |
| --- | --- | --- |
| <1> | Lung (small cell) | CBDCA + ETP |
| <2> | Lung (small cell) | CBDCA + ETP |
| <3> | Lung (small cell) | CBDCA + ETP |
| <4> | Lung (non-small cell) | CBDCA + nab-PTX |
| <5> | Lung (non-small cell) | CBDCA + nab-PTX |
| <6> | Lung (non-small cell) | CBDCA + nab-PTX |
| <7> | Lung (non-small cell) | CBDCA + PAC + Bev |
| <8> | Lung (non-small cell) | CBDCA + PEM |
| <9> | Lung (non-small cell) | CBDCA + PTX + Bev |
| <10> | Lung (non-small cell) | CBDCA + PTX |
| <11> | Lung (non-small cell) | nab-PTX |
| <12> | Lung (non-small cell) | CBDCA + PTX + Bev |
| <13>* | Kidney | Axitinib |
| <14>† | Kidney | Axitinib |
| <15> | Kidney | Axitinib |
| <16>‡ | Kidney | Axitinib |
| <17> | Hypopharynx | CBDCA + 5FU |
| <18> | Oropharynx | CBDCA + 5FU |
| <19>ǁ | Oropharynx | CDDP + 5FU |
| <20> | Salivary gland | CDDP + 5FU |
| <21> | Breast | nab-PTX |

*Corresponded to Case 1 in Table 2 and Case 1 in S1 Table.

†Corresponded to Case 2 in Table 2.

‡Corresponded to Case 4 in Table 2.

ǁCorresponded to Case 9 in Table 2.

5FU, fluorouracil; Bev, bevacizumab; CBDCA, carboplatin; ETP, etoposide; ICI, immune checkpoint inhibitor; PEM, pemetrexed sodium hydrate; PTX, paclitaxel

**S3 Table. Diagnostic Criteria for Myocarditis Reported by the Position Statement of the European Society of Cardiology [8].**

| Myocarditis (WHO /ISFC1): |
| --- |
| Inflammatory disease of the myocardium diagnosed by established histological*, immunological, and immunohistochemical criteria**. |
|  |
| *N.B. established the histological Dallas criteria defined as follows: |
| “histological evidence of inflammatory infiltrates within the myocardium associated with myocyte degeneration and necrosis of non-ischemic origin.” |
| **N.B. unspecified the immunohistochemical criteria; we propose an abnormal inflammatory infiltrate to be defined as follows: |
| “≥14 leucocytes/mm^2^ including ≤4 monocytes/mm^2^ with the presence of CD 3 positive T-lymphocytes ≥7 cells/mm^2^.” |

| Diagnostic criteria for clinically suspected myocarditis |
| --- |
| Clinical presentation |
| Acute chest pain, pericarditic, or pseudo-ischemic |
| New-onset (days up to 3 months) or worsening of the following: dyspnea at rest or exercise and/or fatigue, with or without left and/or right heart failure signs |
| Subacute/chronic (>3 months) or worsening of the following: dyspnea at rest or exercise and/or fatigue, with or without left and/or right heart failure signs |
| Palpitation, unexplained arrhythmia symptoms and/or syncope, and/or aborted sudden cardiac death |
| Unexplained cardiogenic shock |
|  |
| Diagnostic criteria |
| I. ECG/Holter/stress test features |
| Newly abnormal 12 lead ECG finding and/or Holter and/or stress testing as follows: I–III degree atrioventricular block or bundle branch block, ST/T wave change (ST elevation or non ST elevation, T wave inversion), sinus arrest, ventricular tachycardia or fibrillation and asystole, atrial fibrillation, reduced R wave height, intraventricular conduction delay (widened QRS complex), abnormal Q waves, low voltage, frequent premature beats, and supraventricular tachycardia |
| II. Myocardiocytolysis markers |
| Elevated TnT/TnI |
| III. Functional and structural abnormalities on cardiac imaging (echo/angio/CMR) |
| New, otherwise unexplained LV and/or RV structural and functional abnormalities (including incidental finding in apparently asymptomatic subjects): regional wall motion, global systolic, or diastolic function abnormality, with or without ventricular dilatation, increased wall thickness, pericardial effusion, and endocavitary thrombi |
| IV. Tissue characterization by CMR |
| Edema and/or LGE of classical myocarditic pattern |
| Clinically suspected myocarditis if ≥1 clinical presentation and ≥1 diagnostic criteria from different categories were present, in the absence of: (1) angiographically detectable coronary artery disease (coronary stenosis ≥50%); (2) known pre-existing cardiovascular disease or extra-cardiac causes that could explain the syndrome (e.g., valve disease, congenital heart disease, hyperthyroidism, and others). Suspicion is higher with a higher number of fulfilled criteria. |
| If the patient is asymptomatic, ≥2 diagnostic criteria should be met. |

LV, left ventricular; RV, right ventricular; CMR, cardiac magnetic resonance; ECG, Electrocardiography

**S4 Table. Grading of Myocarditis in Common Terminology Criteria for Adverse Events (CTCAE) v5.0 [12].**

| **CTCAE term** | **Grade 1** | **Grade 2** | **Grade 3** | **Grade 4** | **Grade 5** |
| --- | --- | --- | --- | --- | --- |
| Myocarditis | - | Symptoms with moderate activity or exertion | Severe with symptoms at rest or with minimal activity or exertion; intervention indicated; new onset of symptoms | Life-threatening consequences; urgent intervention indicated (e.g., continuous IV therapy or mechanical hemodynamic support) | Death |

IV, intravenous

**S5 Table. Guideline for Diagnosis and Grading of ICI-related Cardiac Toxicity Reported by the American Society of Clinical Oncology (ASCO) [11].**

| **Grading** |  |
| --- | --- |
| G1 | Abnormal cardiac biomarker testing without symptoms and ECG abnormalities |
| G2 | Abnormal cardiac biomarker testing with mild symptoms or new ECG abnormalities without conduction delay |
| G3 | Abnormal cardiac biomarker testing with either moderate symptoms or new conduction delay |
| G4 | Moderate to severe decompensation, IV medication or intervention required, life-threatening conditions |

ECG, electrocardiography; IV, intravenous; ICI, immune checkpoint inhibitor

**S6 Table. Diagnostic Criteria for ICI-related Myocarditis Proposed by Bonaca et al. [7].**

| **Categories of myocarditis** | |
| --- | --- |
| Definite myocarditis | Any of the following: 1. Tissue pathology diagnostic of myocarditis (e.g., on biopsy or autopsy) 2. CMR findings indicating myocarditis, a clinical syndrome, and one of following:  a. Elevated biomarker of cardiac myonecrosis  b. ECG evidence of myo-pericarditis 3. New wall motion abnormality on echocardiography not explained by another diagnosis (e.g., acute coronary syndrome, stress-induced cardiomyopathy, and sepsis) and all the following conditions:  a. Clinical syndrome consistent with myocarditis  b. Elevated biomarker of cardiac myonecrosis  c. ECG evidence of myo-pericarditis  d. Negative angiography or other testing to |
| Probable myocarditis | Any of the scenarios below that are not explained by another diagnosis (e.g., acute coronary syndrome, trauma, and stress-induced cardiomyopathy): 1. CMR findings indicating myocarditis without any of the following:  a. Clinical syndrome consistent with myocarditis  b. Elevated biomarker of cardiac myonecrosis  c. ECG evidence of myo-pericarditis 2. Non-specific CMR findings suggestive of myocarditis with one or more of the following:  a. Clinical syndrome consistent with myocarditis  b. Elevated biomarker of cardiac myonecrosis  c. ECG evidence of myopericarditis 3. New wall motion abnormality on echocardiography with a clinical syndrome consistent with myocarditis and either:  a. Elevated biomarker of cardiac myonecrosis  b. ECG evidence of myopericarditis 4. A scenario meeting the criteria for possible myocarditis (see below) with 18-fluorodeoxyglucose positron emission tomography imaging showing patchy cardiac fluorodeoxyglucose uptake without another explanation |
| Possible myocarditis | Any of the scenarios below that are not explained by another diagnosis (e.g., acute coronary syndrome, trauma, and stress-induced cardiomyopathy): 1. Non-specific CMR findings suggestive of myocarditis with none of the following:  a. Clinical syndrome consistent with myocarditis  b. Elevated biomarker of cardiac myonecrosis  c. ECG evidence of myopericarditis 2. New wall motion abnormality on echocardiography and one of the following:  a. Clinical syndrome consistent with myocarditis  b. ECG evidence of myopericarditis  3. New elevated biomarker (beyond baseline) and one of the following: a. Clinical syndrome consistent with myocarditis b. ECG evidence of myopericarditis |

CMR, cardiac magnetic resonance; ECG, electrocardiography; IV, intravenous

**S7 Table. Cardiac Troponin I Level in Patients With ICI-related Myocardial Damage at Follow-up.**

| **Case no.** | **Pre-ICI** | **1 m** | **2 m** | **3 m** | **4 m** | **5 m** | **6 m** |
| --- | --- | --- | --- | --- | --- | --- | --- |
| 1 | <0.001 | <0.001 | <0.001 | 0.072 | 0.033 | <0.001 | <0.001 |
| 2 | <0.001 | <0.001 | 0.048 | 0.038 | 0.069 | 0.097 | 0.041 |
| 3 | <0.001 | <0.001 | <0.001 | <0.001 | <0.001 | <0.001 | <0.001 |
| 4 | <0.001 | <0.001 | 0.031 | <0.001 | <0.001 | <0.001 | <0.001 |
| 5 | <0.001 | 0.304 | 0.248 | 0.015 | <0.001 | <0.001 | <0.001 |
| 6 | <0.001 | <0.001 | <0.001 | <0.001 | <0.001 | <0.001 | 0.038 |
| 7* | <0.001 | 0.133 | NA | NA | NA | NA | NA |
| 8 | 0.021 | 0.013 | 0.023 | 0.018 | 0.012 | 0.014 | 0.036 |
| 9* | <0.001 | <0.001 | <0.001 | 0.071 | NA | NA | NA |
| 10 | <0.001 | <0.001 | 0.039 | 0.035 | 0.012 | <0.001 | <0.001 |

* Cases 7 and 9 stopped follow-up before 6 months because of the discontinuation of cancer therapy and transition to palliative care.

ICI, immune checkpoint inhibitor; NA, not available.

**S8 Table. NT-proBNP Levels in Patients With ICI-related Myocardial Damage at Follow-up.**

| **Case no.** | **Pre-ICI** | **1 m** | **2 m** | **3 m** | **4 m** | **5 m** | **6 m** |
| --- | --- | --- | --- | --- | --- | --- | --- |
| 1 | 44 | 117 | 61 | 78 | 67 | 45 | 53 |
| 2 | 46 | 84 | 684 | 1374 | 1288 | 1355 | 438 |
| 3 | 604 | 656 | 710 | 1033 | 958 | 725 | 1338 |
| 4 | 291 | 1904 | 4932 | 242 | 59 | 36 | 74 |
| 5 | 37 | 76 | 60 | 54 | 94 | 155 | 91 |
| 6 | 68 | 151 | 112 | 72 | 180 | 178 | 70 |
| 7 | 332 | 522 | NA | NA | NA | NA | NA |
| 8 | 39 | 84 | 45 | 30 | 46 | 75 | 489 |
| 9 | 101 | 106 | 108 | 135 | NA | NA | NA |
| 10 | 55 | 38 | 78 | 98 | 54 | 73 | 48 |

* Cases 7 and 9 stopped follow-up before 6 months because of the discontinuation of cancer therapy and transition to palliative care.

ICI, immune checkpoint inhibitor; NA, not available; NT-proBNP, N-terminal pro-brain natriuretic peptide.

**S9 Table. LVEF in Patients With ICI-related Myocardial Damage at Follow-up.**

| **Case no.** | **Pre-ICI** | **3 m** | **6 m** |
| --- | --- | --- | --- |
| 1 | 72 | 72 | 74 |
| 2 | 68 | 68 | 60 |
| 3 | 57 | 56 | 41 |
| 4 | 54 | 31 | 48 |
| 5 | 69 | 74 | 69 |
| 6 | 79 | 74 | 73 |
| 7 | 67 | NA | NA |
| 8 | 67 | 66 | 67 |
| 9 | 62 | 62 | NA |
| 10 | 67 | 67 | 67 |

* Cases 7 and 9 stopped follow-up before 6 months owing to the discontinuation of cancer therapy and transition to palliative care.

ICI, immune checkpoint inhibitor; LVEF, left ventricular ejection fraction; NA, not available.


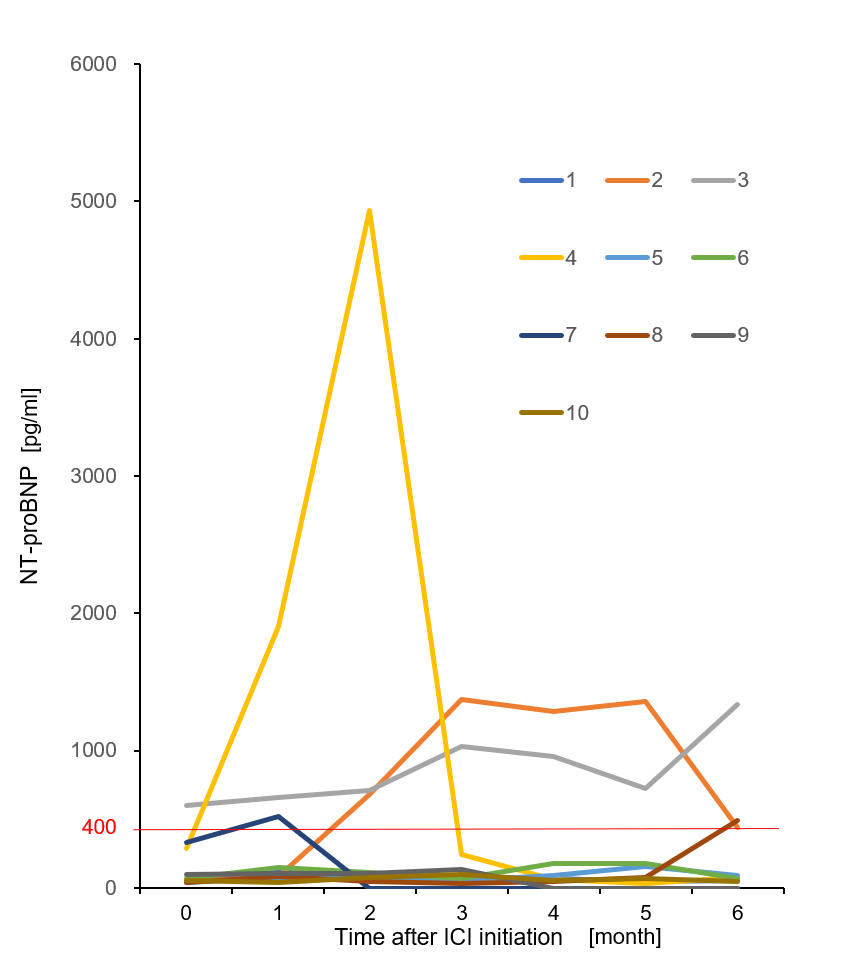


**S1 Fig. NT-proBNP Levels in Patients With ICI-related Myocardial Damage.**

Each patient is indicated with a different color in Fig 1.

Abbreviations: ICI, immune checkpoint inhibitor; NT-proBNP, N-terminal pro-brain natriuretic peptide.


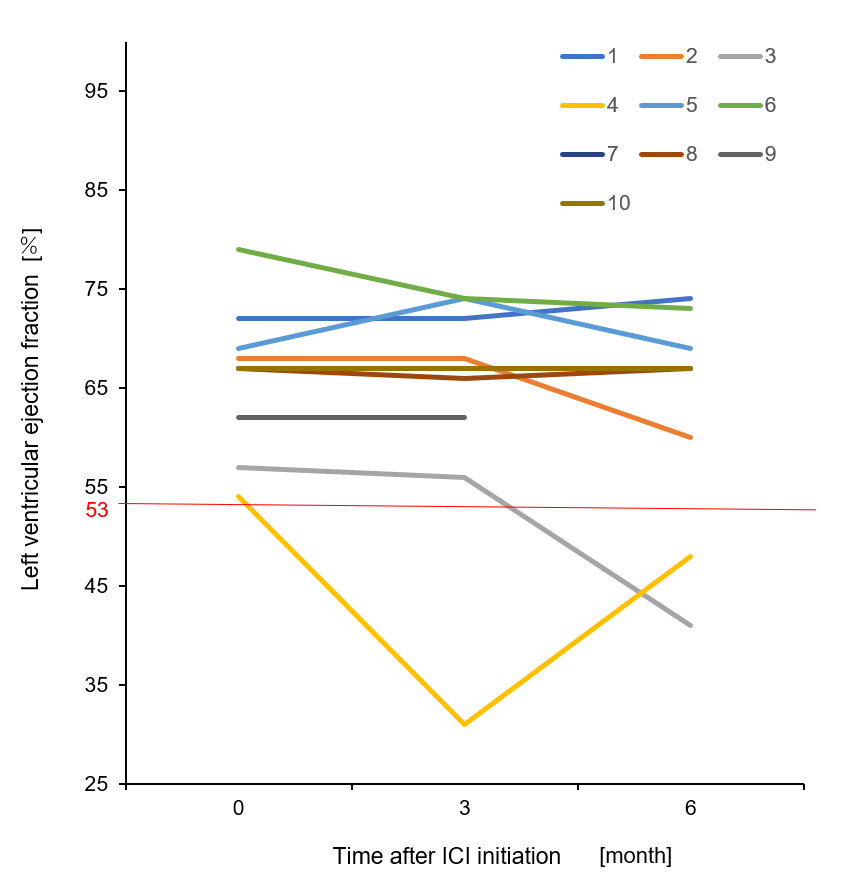


**S2 Fig. Change in the Left Ventricular Ejection Fraction in 10 Patients With ICI-related Myocardial Damage During the Follow-up Period.**

Each patient is indicated with a different color in Fig 1.

Abbreviation: ICI, immune checkpoint inhibitor.

**S1 Method**

**Assessment for shared antigen in cancer tissues**

Among patients diagnosed with myocarditis, those who had sufficient cancer tissue obtained from biopsy or surgery were assessed for shared antigens. In contrast, those who had continued ICI therapy for 6 months without any evidence of irAEs and those who had had sufficient samples of primary cancer taken during surgery were enrolled in the control group. In both groups, total RNA was extracted from cancer samples using the Maxwell RSC RNA FFPE kit (Promega Corp., Madison, WI, USA). The quality of the extracted total RNA was determined using an RNA6000 pico kit (Agilent Technologies, Santa Clara, CA, USA). The gene expression of myocardial proteins in cancer tissues was assessed by RNA sequencing (RNA-Seq). Total RNA from each cancer cell line was prepared using the SMART-Seq Standard Kit and SMARTer RNA Unique Dual Index Kit (Takara, Shiga, Japan). RNA libraries were prepared from at least 0.1 ng (average of 0.8 ng) of total RNA. Total RNA was sequenced on NovaSeq 6000. After mapping the data, gene expression analysis was performed using the DRAGEN Bio-IT Platform (Illumina, San Diego, CA, USA). Depths of 90–150 million paired-end 150 bp reads were generated for each sample.

Next, we analyzed whole genes expressed differently between the control group and the myocarditis group by visualizing them using the heat map function of the R software (R Foundation for Statistical Computing, Vienna, Austria). The differences in the genes were assessed by differential expression analysis using DESeq2 of the R package. Significantly different genes (p<0.05) are shown.

**S10 Table. Baseline Characteristics of Control Patients Used for Expression Analysis by RNA-Seq.**

|  | **Age (years)** | **Sex** | **Cancer location** |
| --- | --- | --- | --- |
| C1 | 68 | Male | Pharynx |
| C2 | 59 | Male | Pharynx |
| C3 | 75 | Male | Pharynx |
| C4 | 86 | Male | Malignant melanoma |
| C5 | 57 | Male | Esophagus |
| C6 | 50 | Male | Lung (non-small cell) |
| C7 | 48 | Male | Lung (non-small cell) |
| C8 | 81 | Male | Lung (non-small cell) |
| C9 | 72 | Male | Esophagus |
| C10 | 44 | Female | Kidney |
| C11 | 52 | Male | Pharynx |
| C12 | 65 | Female | Esophagus |
| C13 | 55 | Female | Fallopian tube |
| C14 | 67 | Female | Malignant melanoma |

Data on age, sex, and cancer types of the 14 control patients are shown in the table.

**
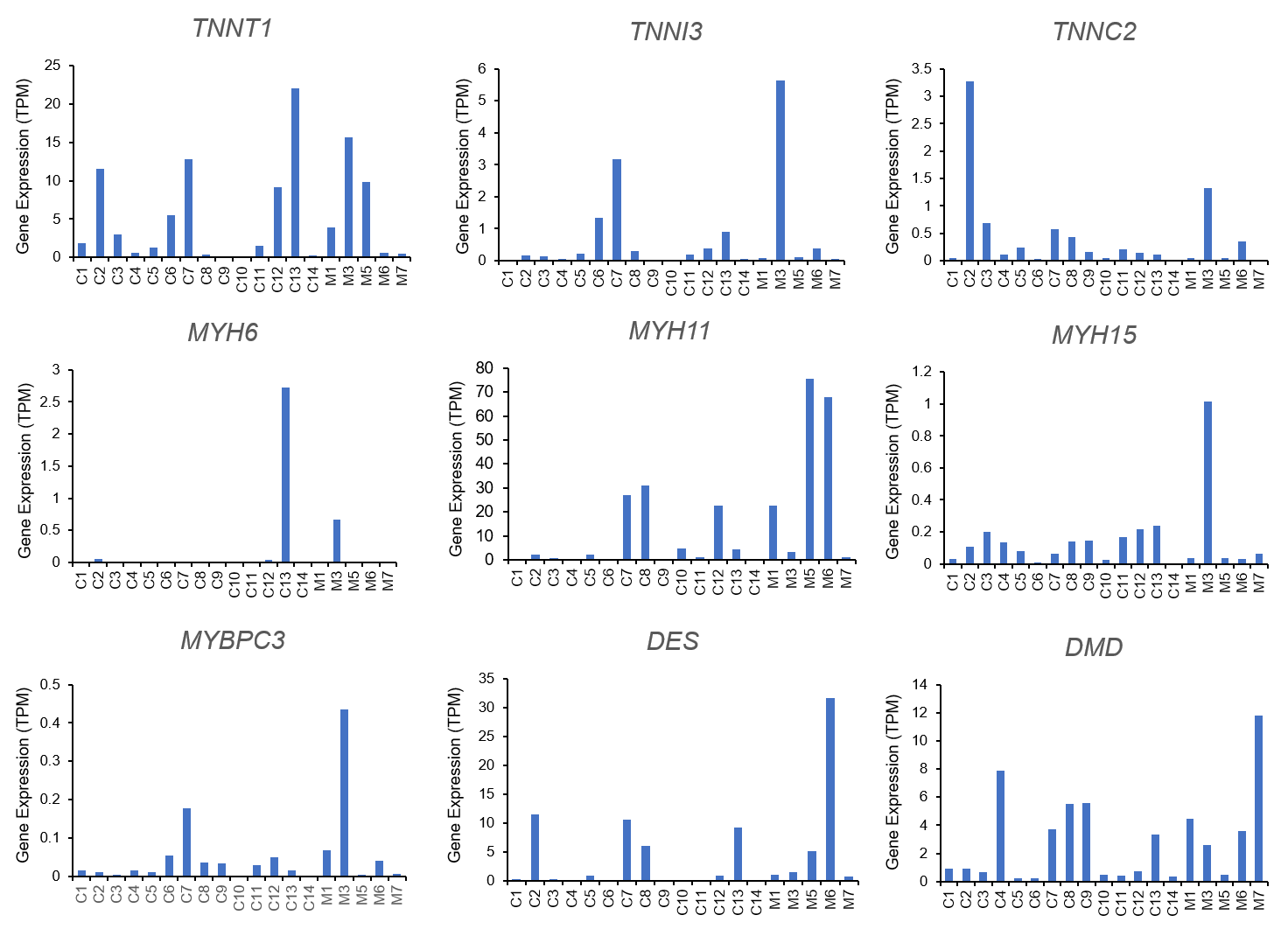
**

**S3 Fig. Expression Analysis Data Obtained From RNA-Seq.**

Expression of cardiomyocyte-related genes in cancer cells was screened for in five patients with myocardial damage (M1–5) and control patients (C1–14). There was no specific pattern for each group. However, those who had myocardial damage seemed to have higher expression of cardiomyocyte-related genes.


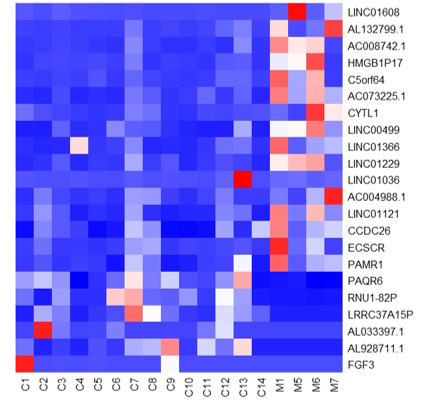


**S4 Fig. Gene Expression Analysis Between the Myocardial Damaged and Control Groups.**

The genes that were significantly differentially expressed are shown in the right row. However, most of them are pseudogenes or non-RNA coding genes. Patient M2 was excluded from this analysis because gene expression was extremely high in most genes.


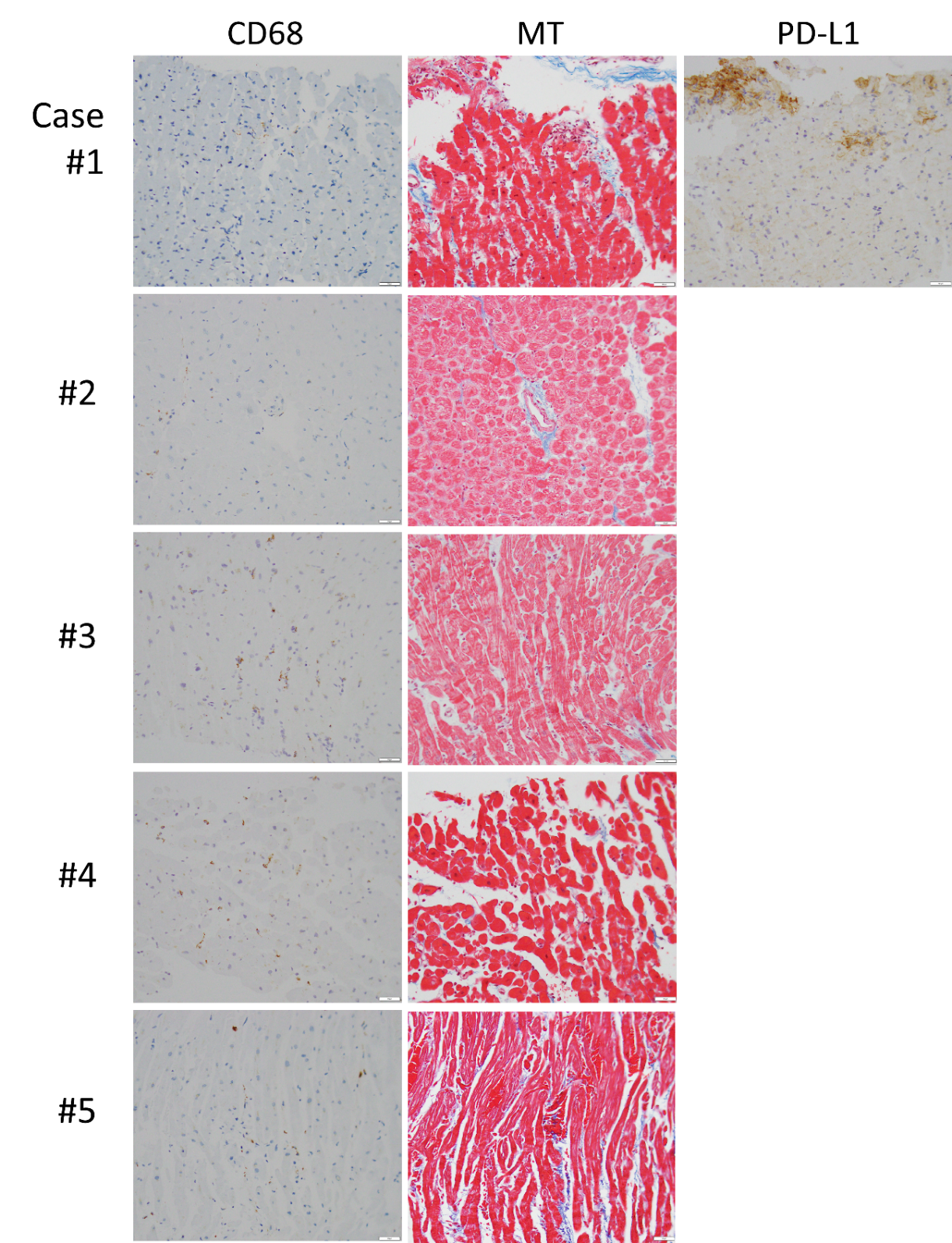


**S5 Fig. Histopathological Study Images of Five Patients With ICI-related Myocardial Damage.**

Sections collected from all patients were stained with CD68 for macrophage and Masson trichrome for fibrosis. In addition, PD-L1 is positive only in Case 1.

Abbreviations: ICI, immune checkpoint inhibitor; MT, Masson’s trichrome; PD-L1, programmed death-ligand 1.
